# Comparative Classes of the Kentucky and Ohio Schools

**Published:** 1834

**Authors:** 


### V.—Comparative Classes of the Medical Schools in Cincinnati and Lexington.

For years, the annual circulars and advertisements of the trustees and faculty of the medical college of Ohio, have set forth to the public, how much the general assembly had done for the benefit of the institution, in providing a hospital for clinical instruction, in furnishing money to erect a splendid edifice, and for the purchase of books, engravings, models, anatomical preparations and chemical apparatus. Even within the last few months a paragraph from one of the *professors*, in a newspaper of the city, gravely announced, that a respectable physician from London, who had visited the college, expressed his gratification in decided terms, and declared that its "*accommodations are far superior to those of several schools in Great Britain, that rank high in that country.*" It would appear, then, that the state of Ohio has made liberal appropriations of money—that there is no deficiency in the *physical* means of instruction—and that the Cincinnati school has, actually, the advantage over that of Lexington in its hospital, and the consequent study of practical and morbid anatomy, as well as of witnessing the treatment of clinical, surgical and obstetrical cases. Being thus, in the apparatus for instruction, even better provided than the Transylvania school, the question naturally arises, why that institution constantly has two or three times as many students as this? If it is owing to any other cause than the superior qualifications and higher standing of the *professors* of that school, that cause ought to be pointed out and abated.

It has often surprised us, that the people of Ohio have not long since opened their eyes to the acknowledged fact, that their school is far inferior in numbers and reputation to that of Kentucky, although their ages are nearly the same, and the expenditures of public money upon the former, have been nearly four times as great as upon the latter; for even the hall in which the lectures are delivered in Lexington, was built by the professors, and some of the capitalists of the city who receive an annual dividend on their loans.

That the people and the physicians of Ohio, may see the comparative numbers in the two institutions from the commencement of their own, we shall present them with the following table, extending through fourteen years. The Lexington school was in operation two sessions before ours, but its two first classes amounted only to 57, two more than were in the two first classes of the Cincinnati school.

TABLE.

| Years. | Cincinnati. | Lexington. |
|---|---|---|
| 1820 | 25 | 93 |
| 1821 | 30 | 138 |
| 1822 | 18 | 171 |
| 1823 | 00 | 200 |
| 1824 | 15 | 234 |
| 1825 | 30 | 281 |
| 1826 | 80 | 190 |
| 1827 | 101 | 152 |
| 1828 | 101 | 206 |
| 1829 | 107 | 199 |
| 1830 | 124 | 210 |
| 1831 | 131 | 215 |
| 1832 | 72 | 222 |
| 1833 | 102 | 262 |
| Total | 936 | 2773 |

It must be borne in mind, that the numbers in the first column, embrace the *beneficiaries*, who have been provided for by the statutes of Ohio. These have been admitted for the last seven years, and have varied in number from 8 or 10 up to 17—the average number about 12, or 84 in the whole period—none of whom it may be presumed, would have attended the lectures, if they had not come in gratis. They should, therefore, be deducted, when the aggregate number, for fourteen years is but 852, while the aggregate of the Lexington school is 2773—giving a difference of more than three to one in favor of the *professors* of the former; although according to the advertisements of the latter, they have labored under several disadvantages, from which the Cincinnati professors were exempt. One of these embarrassments was the higher price and greater number of their tickets—seven at $15, amounting to $105; while the tickets of the Cincinnati professors, varying from four to six in number, and from $10 to $12 in price, amounted only to $40 or $60. Thus, we see, that even underbidding could not keep our institution from being distanced! How it may hold on in future, we shall not predict; but as the faculty have lately advertised for an indefinite number of beneficiaries, to supply the places of those lost by a change in the policy of the state in this respect, they will probably be able to keep up what for *them* will be a respectable number; and thus do away the necessity of abolishing another professorship for the purpose of cheapening the institution. It is, moreover, a favorable symptom, that the professors require the applicant to *swear*, that his application "*is in his own composition and hand writing*," as by this precaution none will hereafter make their way into the college, who have not learned to write. Should the faculty persevere in *such* labors for raising the character of the institution, it will soon attain an elevation to which the *cross*-bow of calumny cannot send its shafts.

